# Will centralized drug procurement policy improve enterprises’ total factor productivity?

**DOI:** 10.3389/fpubh.2025.1504342

**Published:** 2025-07-02

**Authors:** Xin Li, Ran Tao, Wenxue Zou, Yuning Jin

**Affiliations:** ^1^Capital University of Economics and Business, Beijing, China; ^2^Financial Interbank Department, Beijing Rural Commercial Bank, Beijing, China

**Keywords:** national centralized drug procurement, listed drug manufacturing enterprises, healthcare policy reform, financing constraints, total factor productivity

## Abstract

Against the backdrop of China’s healthcare reform challenges in drug pricing, this study investigates the impact of the National Centralized Drug Procurement (NCDP) policy implemented in 2018. Employing Difference-in-Differences (DID) methodology on quarterly data from A-share listed pharmaceutical firms (2003-2021, sourced from WIND/CSMAR databases), we demonstrate that NCDP participation significantly reduces Total Factor Productivity . Robustness is confirmed through index substitution, propensity score matching, and lag tests. The negative effect is amplified in non-state-owned enterprises, non-TCM manufacturers, and firms with high analyst coverage. Mechanistically, NCDP suppresses TFP through: (i) tightened financing constraints (↑KZ index) impairing capital allocation efficiency, and (ii) an indirect pathway where short-term R&D surges (↑RD) trigger resource crowding-out effects, compounded by diminished investment efficiency (↑INV), ultimately forming an “R&D→investment inefficiency→TFP↓” transmission chain. To reconcile public welfare objectives with corporate sustainability, we propose dual optimization strategies: *differentiated financing support* and *innovation incentive reform*. These establish a sustainable equilibrium between price control and TFP enhancement, providing actionable solutions for nationwide NCDP scaling.

## 1 Introduction

Excessively inflated drug prices have long been a persistent issue in China’s healthcare reform. Despite ongoing policy efforts to reduce residents’ medical expenses, the results have been minimally effective. The primary reason is that these policies fail to address the strategies used by hospitals and pharmaceutical companies to circumvent and undermine their effects, leading to limited success in lowering drug prices. Consequently, problems such as “decoupling of quantity and price,” “fragmented procurement,” and “financing medical services through drug sales” remain unresolved. To address these issues, relevant authorities have gradually implemented the National Centralized Drug Procurement (NCDP) reform.

The NCDP policy, administered by the National Healthcare Security Administration (NHSA), seeks to substantially lower drug procurement costs by requiring designated medical institutions to allocate most of their purchase volumes to winning bidders. This approach, based on the principles of “volume-for-price exchange” and “quantity-price linkage,” imposes dual pressures on pharmaceutical manufacturers by simultaneously affecting product pricing and production volumes. As the policy undergoes further refinement, systematically examining its actual impact on the Total Factor Productivity (TFP) of pharmaceutical firms is crucial for evaluating its effectiveness and informing institutional design improvements. Adopting a corporate finance perspective, this study explores how NCDP affects firm-level TFP and proposes institutional improvements to support collaborative governance under the NCDP framework.

Existing research on NCDP has primarily examined its effects on price control. Empirical evidence confirms that the policy significantly reduces the prices of drugs listed in the procurement catalog, with the extent of price suppression negatively correlated with market concentration (1–4). However, the micro-level mechanisms through which NCDP influences pharmaceutical firms remain insufficiently explored. As an institutional innovation in public procurement, NCDP aligns with demand-side industrial policy tools (5), offering a theoretical framework for analyzing its firm-level effects. TFP, a core indicator of high-quality enterprise development, serves as a key mechanism through which industrial policy promotes economic transformation and upgrading. This study empirically examines the impact of NCDP on enterprise TFP using microdata from pharmaceutical manufacturers, aiming to uncover how demand-side industrial policies influence corporate performance and productivity.

To address these questions, the study employs quarterly data from China’s A-share listed pharmaceutical manufacturing firms between 2003 and 2021. A multi-period difference-in-differences (DID) approach is used to assess the causal impact of NCDP on TFP. Additionally, the study investigates heterogeneity in NCDP’s effects across firms with different characteristics and explores the transmission mechanisms through which the policy influences firm productivity.

This study makes three key contributions. First, it expands the understanding of NCDP’s microeconomic effects by focusing on enterprise TFP, providing empirical evidence on how centralized procurement participation shapes firm productivity. Second, by leveraging the capital market’s resource allocation functions, it identifies specific mechanisms—such as heightened financing constraints, increased innovation investment, and reduced investment efficiency—through which NCDP affects productivity, revealing chain-mediated mechanisms that enhance understanding of the interaction between macro-level regulation and micro-level corporate efficiency. Third, the study analyzes heterogeneous effects based on ownership structure, product type, and analyst coverage, offering practical insights for refining healthcare market reforms, optimizing policy design, and improving enterprise TFP, thereby supporting evidence-based policymaking and collaborative implementation under the NCDP framework.

## 2 Policy background, research status, and research hypotheses

### 2.1 Institutional background of NCDP

NCDP is a reform led by the NHSA where the bidding department commits to allocating most of the procurement share from designated medical institutions to the winning companies. This approach aims to reduce drug prices by leveraging bulk purchasing. The concepts of “exchanging volume for price” and “linking quantity with price” reflect this strategy. By consolidating demand from multiple provinces and cities, a “joint procurement office” is established to implement the NCDP on behalf of public medical institutions. The procurement process starts with selecting NCDP drugs. Government departments then collate drug demand from medical institutions and determine the planned NCDP volume. Based on this, the Medical Insurance Bureau meets with pharmaceutical companies to attract bids and eventually announces the volume-based procurement plan (6).

Since 2015, China has introduced successive drug procurement policies such as the “Opinions on Further Regulating the NCDP Work of Medical Institutions” and the “Guidance on Improving the NCDP Work of Public Hospitals,” aiming to coordinate the reform of drug procurement processes and bidding methods. Through the principle of “linking quantity and price” and competitive bidding, the NCDP establishes prices by exchanging quantity for price.

The primary distinction between NCDP and previous bidding procurements is that NCDP announcements commit winning enterprises to procure the majority share of specified drug types. Drugs not included in NCDP can still be procured through regular bidding, but their market share is reduced. Higher procurement volumes incentivize pharmaceutical companies to lower bid prices, facilitating quantity-for-price exchange. Moreover, after signing contracts, hospitals swiftly execute procurement plans to avoid penalties. The Medical Insurance Bureau oversees drug procurement, minimizing hospitals’ opportunities for secondary price negotiations and kickbacks. By adhering to NCDP volume commitments, drug procurement costs are effectively reduced.

In January 2021, the State Council issued the “Opinions on Promoting the Normalization and Institutionalization of NCDP Work” (referred to as the “opinions” hereafter). These “opinions” advocate for enhancing a market-led drug price formation mechanism, harnessing the strategic purchasing power of the medical insurance fund, and advancing the normalization and institutionalization of NCDP initiatives. The objective is to establish a governmental organizational framework, alliance procurement strategies, and platform operations, accelerating the establishment of a unified and accessible NCDP market nationwide. This aims to guide drug prices towards reasonable levels, significantly alleviate the financial burden of medications on the public, foster healthy growth in the pharmaceutical sector, drive reforms in public medical institutions, and ensure equitable access to essential healthcare services.

Regarding NCDP categories, the emphasis is on including drugs from the basic medical insurance drug catalog that are essential for clinical needs and have high usage and procurement costs. The objective is to progressively encompass all domestically listed drugs that are clinically necessary and of reliable quality, achieving comprehensive procurement. Priority is given to drugs that have undergone (or are deemed to have undergone) consistency evaluation for generic drug quality and efficacy (referred to as “consistency evaluation”).

Regarding procurement rules, the agreed procurement ratio is determined reasonably based on the drug’s clinical usage characteristics, market competition patterns, and the number of selected enterprises. This ratio is maximized under the premise of ensuring quality, maintaining supply, and preventing monopolies. The selection of enterprises is based on market competition and supply capacity, emphasizing scale effects and fostering effective competition. Participation by enterprises is voluntary, with independent quoting. Selections are made through competition on quality and price. The outcomes reflect the principle of linking quantity and price, clearly defining the agreed procurement volumes for each selected enterprise.

To manage the NCDP budget effectively, a prepayment mechanism is established where the medical insurance fund pre-pays no less than 30% of the annual agreed procurement amount to medical institutions. As institutions progress in procurement, this prepayment is gradually offset against medical expenses claimed.

To date, NCDP has successfully conducted eight rounds of bidding activities covering over 300 drug categories.

NCDP becomes increasingly standardized and institutionalized, it has disrupted the non-equilibrium structure of the government pricing model, allowing multiple entities to participate more comprehensively in the entire drug pricing process under established rules. NCDP, functioning as a government procurement strategy, essentially operates as a demand-side industrial policy tool. However, its notable distinction from previous demand-side industrial policy tools lies in its additional conditions, which set product prices significantly below prevailing market levels while requiring substantial product supply. Therefore, NCDP drug pricing itself represents a distinctive form of demand-side industrial policy.

Pharmaceutical manufacturing companies, as market participants, are inevitably influenced by both the combined impacts of product prices and production volumes. As policies progressively refine, the effects on these companies necessitate thorough investigation. Therefore, this paper explores the actual impact of NCDP on firms’ TFP from a corporate finance perspective. We propose institutional improvements aimed at fostering a cooperative and mutually beneficial outcome for all stakeholders within the NCDP framework.

### 2.2 Industrial policy and TFP

Kenneth Arrow and Gérard Debreu’s General Equilibrium Theory provides a foundational framework for analyzing interactions among market variables within economic systems. Within this framework, market prices for all goods and services are endogenously determined through supply and demand interactions, leading to optimal resource allocation and market equilibrium. When the government imposes price ceilings below market equilibrium and engages in large-scale procurement, these artificial controls disrupt normal supply–demand dynamics and result in price distortions.

Under the NCDP policy, the government functions as the primary buyer, with procurement expenditures directly flowing to enterprises. This mechanism directly influences firms’ production, operations, and investment decisions, and ultimately impacts their production efficiency. By setting prices below market equilibrium and implementing bulk procurement, the NCDP policy results in three primary efficiency losses: first, distorted price signals hinder efficient resource allocation and suppress corporate innovation (7); second, non-competitive procurement leads to adverse selection, enabling inefficient firms to obtain contracts via rent-seeking, which results in crowding-out effects (8, 9); third, excessive reliance on procurement orders reduces market adaptability—evidence from Chinese enterprise data indicates that a 1% increase in government procurement share leads to a 0.5% decline in TFP (10). Nakabayashi (11) further shows that preferential procurement policies substantially raise transaction costs and diminish market efficiency.

From a demand-side industrial policy perspective, the NCDP theoretically generates a “demand-pull” effect that could stimulate corporate innovation (12). However, empirical findings are mixed: Aghion et al. (13) report that competition-enhancing policies increase TFP, whereas Qian et al. (14) find that the Ten Key Industry Revitalization Plans reduced firm productivity. Zhang et al. (15) also demonstrate that targeted industrial policies inhibit TFP growth, while inclusive policies facilitate more efficient resource allocation (16). This divergence arises from variations in policy instruments—NCDP, as a non-standard policy tool, combines ultra-low price constraints with centralized procurement, potentially creating unique channels of influence. Although improving the institutional environment can enhance TFP by mitigating contractual risks (17, 18), administrative intervention in implementation may still lead to resource misallocation (19).

While prior studies generally acknowledge that government procurement influences firm-level TFP, the direction and magnitude of this impact remain debated due to heterogeneity in policy tools and firm characteristics. For the NCDP in particular—characterized by a combination of price regulation and demand stimulation—its micro-level transmission mechanisms warrant further empirical investigation.

*Hypothesis 1*: The NCDP policy negatively affects the TFP of Chinese pharmaceutical manufacturers.

### 2.3 Heterogeneity of industrial policy’s impact on TFP

Recent studies reveal substantial heterogeneity in the effects of industrial policies on firms’ TFP, driven by the interplay of policy instruments, industry characteristics, and firm-level attributes. Across policy instruments, different tools—such as subsidies, tax incentives, and factor price controls—affect firms through distinct transmission channels. Zhao and Lin (20) argue that price-control-based industrial policies exhibit greater sensitivity to firm size heterogeneity, while Cao and Xia (21) find that competition-oriented industrial policies disproportionately benefit small and medium-sized enterprises (SMEs) relative to large firms.

At the industry level, labor-intensive sectors are significantly more responsive to local industrial policy initiatives than capital- or technology-intensive industries (22, 23), indicating that variations in factor intensity constitute key boundary conditions for divergent policy effects.

In terms of firm heterogeneity, state-owned enterprises (SOEs) enjoy structural advantages in accessing policy resources, stemming from their political ties (15). Moreover, differences in firm lifecycle stages and ownership structures lead to asymmetric responsiveness to policy interventions (24). Market attention further amplifies disparities in policy outcomes: analyst coverage enhances TFP through improved information discovery, with this effect varying significantly across firms (25–27).

*Hypothesis 2*: The impact of NCDP on TFP varies depending on firms’ product types, ownership structures, and levels of market attention.

### 2.4 The impact mechanism of industrial policy on TFP

Prior research has extensively explored the mechanisms through which industrial policies influence TFP, revealing a complex web of transmission mechanisms. From the lens of financing constraints, industrial policies channel capital to firms through fiscal subsidies, credit support, and other instruments, which exert dual effects on TFP by easing capital constraints. On one hand, these policies act as implicit guarantees via signaling mechanisms, lowering external R&D financing costs—particularly in high R&D-intensive industries such as equipment manufacturing and pharmaceuticals—thereby boosting TFP (28, 29). On the other hand, subsidies can distort resource allocation, undermine aggregate manufacturing productivity, and erode market competition efficiency over time (30). This contradiction is especially pronounced in strategic emerging industries, where subsidies promote TFP growth for non-state-owned enterprises (31), but recurring monetary policy shocks exacerbate credit misallocation in favor of state-owned firms, ultimately diminishing investment efficiency for private enterprises (32). This underscores the moderating role of financing constraints as a core mediating variable.

R&D investment constitutes another critical channel. Industrial policies directly stimulate R&D through subsidies and tax incentives; for example, policies targeting strategic emerging industries significantly enhance innovation spending and drive TFP growth (33). Digital firms also capitalize on the signaling value of subsidies to attract external funding (34). At both firm and sectoral levels, R&D improves productivity by optimizing capital-labor allocations (35) and facilitating the reallocation of resources to more efficient industries (36). However, excessive policy intervention can incentivize excessive R&D engagement—particularly in monopolistically competitive sectors like pharmaceuticals—where firms sustain innovation through policy compensation to maintain their technological edge (16). Absent complementary regulatory measures, such interventions may crowd out productive investment, resulting in a trade-off between innovation intensity and investment efficiency (37).

The mediating role of investment efficiency predominantly follows a suppression logic. Industrial-policy-induced expansion often leads to overinvestment and excess capacity (38), while government subsidies further distort capital allocation by crowding out private investment (63). Empirical evidence supports this view, showing that key industrial policies may inhibit TFP growth by misallocating production factors (15). Notably, the interaction between financing constraints and investment efficiency amplifies policy heterogeneity: in credit-dependent sectors, non-state-owned enterprises endure a “double burden” of credit crowding-out—restricted access to financing constrains R&D capabilities and simultaneously suppresses efficient investment, leading to stagnated TFP growth (32).

**Figure 1:**
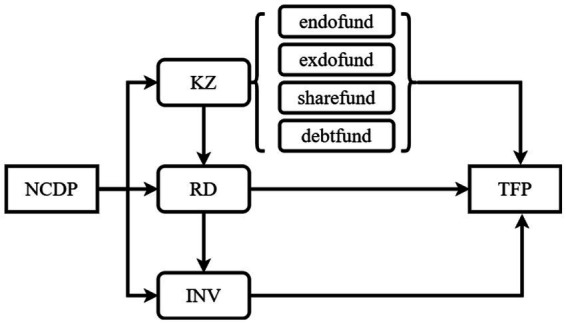
Chain-mediation effect pathway.

In summary, industrial policies influence TFP not through a single linear pathway but via a dynamic chain of interrelated channels: financing constraints → R&D investment → investment efficiency. The positive transmission chain unfolds as follows: policy-induced capital easing → R&D acceleration → improved investment structure → TFP enhancement (39). Conversely, a negative feedback loop arises when credit misallocation suppresses market competition, encourages inefficient investment, and ultimately hinders TFP (14). These coexisting mechanisms call for policy designs that are sensitive to sector-specific attributes—such as the R&D dependence of pharmaceuticals and overcapacity risks in capital-intensive industries—and firm characteristics, especially the financial fragility of private enterprises. Dynamic optimization of policy tools, such as differentiated subsidy phase-out mechanisms, is crucial for balancing productivity gains with efficient resource allocation. The theoretical framework is illustrated in Figure 1.

*Hypothesis 3*: The NCDP will affect firms’ TFP through a chain-mediation mechanism involving financing constraints, innovation investment, and investment efficiency.

## 3 Data and research design

### 3.1 Data sources

Since the inception of the “4 + 7” NCDP pilot in 2019, the types of drugs included in NCDP contracts, the amounts of NCDP funds, and information about pharmaceutical manufacturing enterprises have been publicly disclosed during each bidding process. The procurement data for NCDP contracts come from the Shanghai Sunshine Pharmaceutical Procurement Network, where details about the winning pharmaceutical manufacturers, the unit prices of the winning products, the categories of the winning drugs, and the quantities of the winning drugs procured are disclosed. Additionally, listed pharmaceutical manufacturing companies that win bids will release announcements post-bid, detailing information such as the batch of the winning drugs, the names of the winning products, the sales volume of the winning drugs, the proportion of the winning drugs’ sales volume to the total annual sales of the previous year, and whether the bid win will impact the normal production and operation of the company.

Additional data for this study are sourced from the WIND and CSMAR databases, encompassing quarterly observations of A-share listed pharmaceutical manufacturing firms in China from 2003 to 2021. The use of quarterly data is methodologically justified for several reasons. First, considering that the NCDP policy had been in effect for only three years by the end of the sample period, quarterly frequency enhances statistical power and is better suited for identifying short-term policy effects (40). Second, as shown in Table 1, NCDP bidding rounds occur multiple times annually, which makes quarterly data more precise than annual aggregates in capturing policy shocks (41). Accordingly, this study adopts quarterly data for all regression analyses. Firms designated as ST or *ST—indicating financial distress and potential delisting risk—are excluded from the sample. The final dataset consists of 310 listed firms with complete and continuous reporting, ensuring data consistency and representativeness.

**Table 1:** Overview of NCDP organization.

Batch	Bid opening date	Number of procured varieties	Selection mechanism	Agreed procurement ratio	Criteria for determining winning enterprises	Institutional progress
“4 + 7” pilot	2018-12-17	25	Single winner	100%	The lowest bidding enterprise enters price negotiation.	First national-level pilot organization for drug NCDP.
Pilot expansion	2019-09-30	25	Multiple winners (≤3)	50–70%	The lowest bidding three enterprises confirm supply regions.	First national organization of a 25-province NCDP alliance.
Second batch	2020-01-21	32	Multiple winners (≤6)	50–80%	After meeting the 1.8x circuit breaker mechanism or 50% reduction protection mechanism or 0.1 yuan protection mechanism, the lowest bidding *n* enterprises confirm supply regions. *n* = maximum number of shortlisted enterprises.	Comprehensive national organization of drug NCDP; procurement documents emphasize legal responsibilities and gradually implement policies for retention of surplus medical insurance funds and a credit evaluation system.
Third batch	2020-08-24	55	Multiple winners (≤8)	40–70% for some drugs (antibiotics, hormones, etc.); others: 50–80%		
Fourth batch	2021-02-08	45	Multiple winners (≤10)			
Fifth batch	2021-06-28	61	Multiple winners (≤10)			
Sixth batch	2021-11-26	6	Multiple winners (≤8)		After meeting the 1.3x circuit breaker mechanism or 50% reduction protection mechanism, *n* = maximum number of shortlisted products.	Special procurement for insulin, with agreed procurement volume allocated based on ranking order.
Seventh batch	2022-07-13	61	Multiple winners (≤10)	50–80%	Same as Fifth Batch	Same as Fifth Batch
Eighth batch	2023-02-17	40	Multiple winners (≤10)	30–80%	Same as Fifth Batch	Same as Fifth Batch

### 3.2 Dependent variable

TFP reflects the average output level per unit of input in the production process, representing the overall efficiency of converting inputs into final outputs (42). Existing studies typically measure TFP by deducting the growth rates of input factors from output growth rates (43). Common methods for estimating firm-level TFP include the Olley-Pakes (OP) method (64), the Levinsohn-Petrin (LP) method (44), and ordinary least squares (OLS).

While OLS is the most basic method for estimating TFP, it suffers from two biases: simultaneity bias and selection bias. Due to these statistical limitations, this study primarily employs the LP semi-parametric method to estimate firm-level TFP. Following Levinsohn and Petrin (44) and Lu and Lian (42), the estimation is based on firm revenue, number of employees, and capital expenditures, using cash payments for raw materials and services as a proxy for intermediate inputs in place of the investment variable proposed in the LP method. This approach allows for the estimation of firm-level TFP using the Levinsohn-Petrin productivity estimation procedure. Additionally, since the OP method effectively addresses simultaneity and selection biases, this study also uses OP-estimated TFP (TFP-OP) as an alternative dependent variable. Both LP and OP measures are used to represent firm-level TFP.

### 3.3 Explanatory variable

To estimate the impact of NCDP on the TFP of pharmaceutical manufacturing enterprises, the most direct method is to compare the TFP of pharmaceutical manufacturing enterprises included in the NCDP list with those not included. However, this difference may be influenced by general factors that vary over time, in addition to the impact of NCDP. Since NCDP and recognition occur in multiple periods and at different times, to exclude other interfering factors, this paper uses the multi-period difference-in-differences (DID) method to test the impact of NCDP on the TFP of pharmaceutical manufacturing enterprises. The multi-period DID method can test whether there is a significant difference in the impact on TFP between participating and non-participating enterprises before and after NCDP, controlling for other factors. As this paper uses multi-period panel data, we refer to Yue and Ye (65), and the Model (1) is set as follows:


(1) $$\mathrm{TF}P_{\mathrm{it}}=\beta_{0}+\beta_{1}\text{treatNCDP}*+\beta_{2}\text{contro}l_{\mathrm{it}}+\sum \text{Time}+\sum \text{Prov}+\varepsilon_{\mathrm{it}}$$

Subscripts *i* and *t* denote the firm and quarter, respectively. The key explanatory variable, treatNCDP∗, is constructed as a multi-period difference-in-differences (DID) indicator. Following Cohen et al. (45), firm participation in the NCDP is identified through bidding contracts and publicly disclosed announcements of winning bids by listed firms. Specifically, if firm *i* wins an NCDP contract in quarter *t*, treatNCDP∗ is set to 1 from that quarter onward and 0 for all preceding periods. For firms that never secure an NCDP contract, treatNCDP∗ remains 0 for the entire sample period.

The vector $\text{contro}l_{\mathrm{it}}$ denotes a set of control variables that vary by firm and quarter and are expected to influence TFP. Time and Prov represent time and province fixed effects, respectively, accounting for macroeconomic fluctuations and regional heterogeneity. Firm fixed effects are excluded to preserve degrees of freedom, avoid inflation of standard errors, and maintain the statistical power necessary to detect meaningful associations. The error term $\varepsilon_{\mathrm{it}}\;$captures random disturbances.

### 3.4 Control variables

Drawing on existing literature, this study incorporates control variables from two dimensions—firm operational characteristics and corporate governance structure—to address potential confounding effects. On the operational side, we include the logarithm of firm size, tangible asset ratio, management expense ratio, and sales growth rate to control for heterogeneity in resource endowments, asset composition, operational efficiency, and market expansion capacity. Regarding corporate governance, we include the executive shareholding ratio, separation of ownership and control, proportion of independent directors, and equity balance ratio to capture the effects of managerial incentives, control rights distribution, board supervision, and shareholder oversight on firms’ strategic behavior and resource allocation. Detailed definitions and measurement methods of all variables are provided in Table 2.

**Table 2:** Descriptive statistics.

Variable type	Variable symbol	Variable name	Mean	Std. Dev.	Min	Max
Dependent variables	Tfp_lp	TFP calculated using LP method	6.561	1.053	0.315	8.867
	Tfp_op	TFP calculated using ACF method	6.468	1.039	0.285	8.764
Core explanatory variable	treatNCDP	NCDP dummy variable	0.037	0.188	0.000	1.000
Firm-level control variables	Size	Firm size	21.707	1.013	17.813	24.271
	ESR	Executive shareholding ratio	11.164	17.989	0.000	64.523
	TA	Tangible assets	3.230	2.660	0.000	14.414
	MER	Management expense ratio	0.102	0.053	0.005	0.289
	STR	Separation of two rights	5.986	7.958	−21.590	29.829
	PID	Proportion of independent directors	36.641	5.047	0.000	50.000
	EBD	Equity balance degree	0.726	0.582	0.006	2.600
	SGR	Sales growth rate	0.341	0.695	−1.000	1.897

## 4 Results

### 4.1 Main regression results

Table 3 presents the effects of NCDP implementation on the TFP of pharmaceutical manufacturing firms. Columns (1) and (3) report regression coefficients without control variables, while controlling for time and location effects; none of these coefficients are statistically significant. Only after including enterprise-level variables and applying fixed effects for both time and location do the regression coefficients in Columns (2) and (4) become statistically significant. We suggest that the initial lack of significance is due to the error term, ε_it, incorporating unexplained variations when other factors influencing the dependent variable are not controlled, thereby inflating standard errors and obscuring statistical significance. After including the control variables, the results indicate that NCDP implementation has a statistically significant negative impact (at the 1% level) on the TFP of listed pharmaceutical firms. Based on these findings, it is clear that firms in the treatment group experienced significantly greater declines in TFP compared to the control group following the implementation of NCDP.

**Table 3:** Results of main sample regression.

	(1)	(2)	(3)	(4)
	tfp_lp	tfp_lp	tfp_op	tfp_op
treatNCDP	0.004 (0.053)	−0.183*** (0.039)	−0.046 (0.050)	−0.198*** (0.039)
Size		0.515*** (0.010)		0.460*** (0.010)
ESR		0.006*** (0.000)		0.006*** (0.000)
TA		0.143*** (0.004)		0.141*** (0.004)
MER		−3.966*** (0.190)		−3.898*** (0.189)
STR		0.003*** (0.001)		0.003*** (0.001)
PID		−0.010*** (0.002)		−0.010*** (0.002)
EBD		0.030** (0.014)		0.044*** (0.015)
SGR		0.147*** (0.045)		0.154*** (0.045)
_cons	6.561*** (0.009)	−4.448*** (0.231)	6.469*** (0.009)	−3.374*** (0.231)
Time fixed effects	Control	Control	Control	Control
Province fixed effects	Control	Control	Control	Control
Observations	9,634	9,634	9,634	9,634
R-squared	0.310	0.563	0.322	0.549

Robust standard errors are in parentheses. *** *p* < 0.01, ** *p* < 0.05, * *p* < 0.1.

### 4.2 Robustness tests

#### 4.2.1 Parallel trend test

For the purpose of this study, the difference-in-differences (DID) method requires that the TFP of firms in the treatment and control groups exhibit a parallel trend before the policy shock. Figure 1 depicts the estimated results of $\beta_{1}$ with a 95% confidence interval. It is observed that $\beta_{1}$ is not significant for the eight quarters preceding the policy shock, indicating that there were no substantial differences between the treatment and control groups before the implementation of the NCDP pilot policy. This observation satisfies the parallel trend assumption.

**Figure 2:**
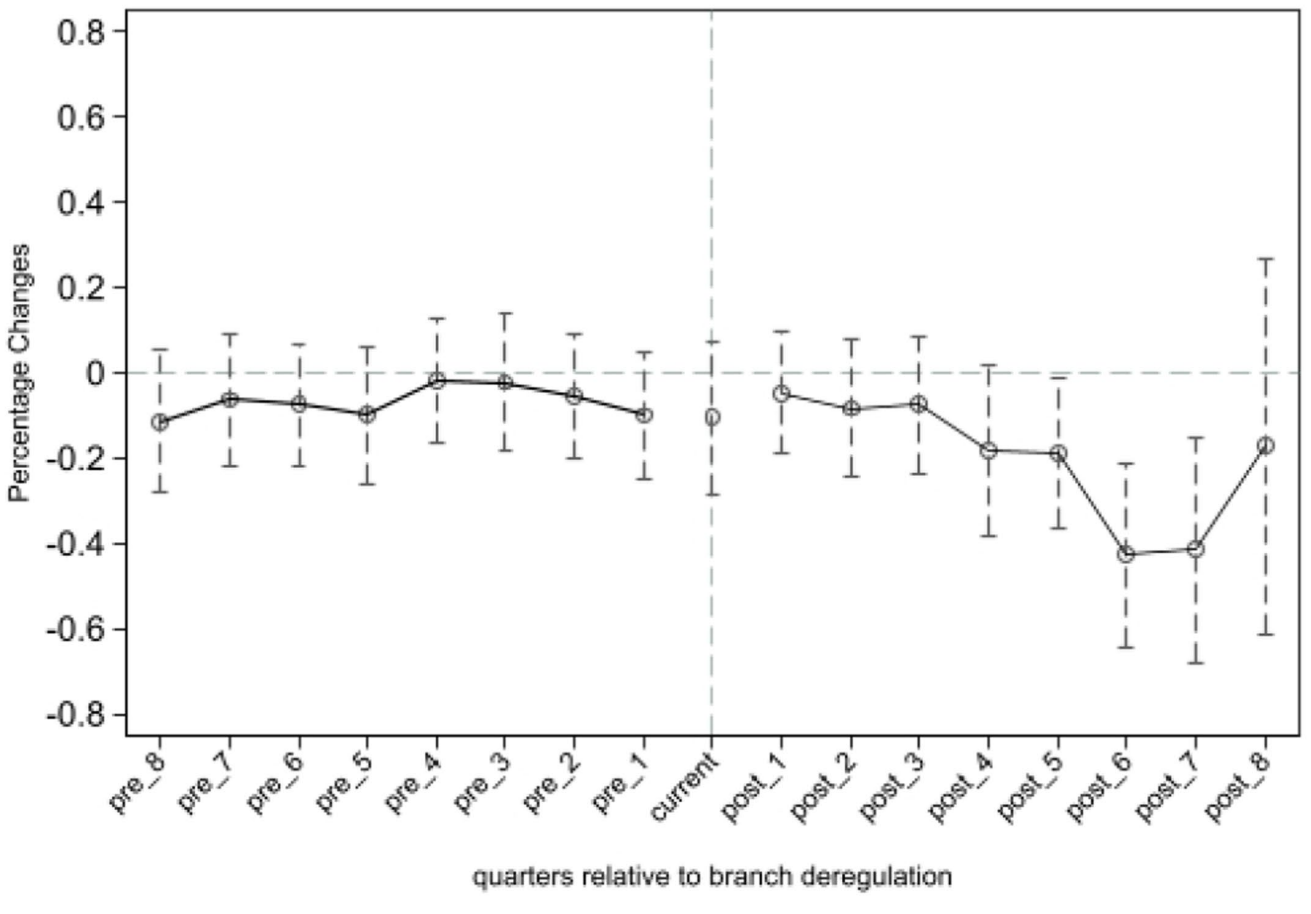
Parallel trend test.

Furthermore, post-implementation, the estimated coefficient $\beta_{1}$ becomes significant and negative starting from the fifth quarter. This indicates that the NCDP has a negative impact on the TFP of the firms included in the NCDP list. Thus, the implementation of the NCDP has lowered the TFP of pharmaceutical manufacturing firms under its purview (see Figure 2).

#### 4.2.2 Alternative regression method

**Figure 3:**
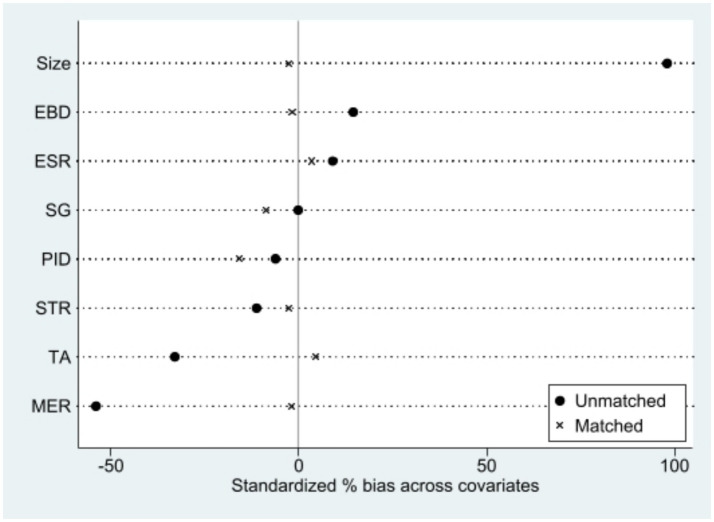
Propensity score distribution after matching.

To test the robustness of the baseline regression results, this study employs the Propensity Score Matching-Difference-in-Differences (PSM-DID) method. Considering that the effects of the NCDP pilot policy were already evident in 2018, this study conducts annual propensity score matching only for samples before the 2018 policy impact. The control variables are used as covariates for annual propensity score matching, retaining only those sample points that fall within the common support range in each matching quarter (Figure 3). A multi-period difference-in-differences test is then conducted exclusively on these samples within the common support range. As shown in the first column of Table 4, the coefficients of the multi-period DID variable are significantly negative at the 1% level. Therefore, the baseline regression results of this study are robust.

**Table 4:** Robustness tests.

	(1) PSM-DID	(2) Time Change DID	(3) L.tfp_lp	(4) L2.tfp_lp	(5) tfp_ols
treatNCDP	−0.607*** (0.155)	−0.169*** (0.038)	−0.188*** (0.037)	−0.161*** (0.039)	−0.141*** (0.036)
_cons	−2.275 (1.378)	−4.144*** (0.285)	−4.385*** (0.228)	−4.656*** (0.245)	−1.569*** (0.219)
Controls	Yes	Yes	Yes	Yes	Yes
Time fixed effects	Controls	Controls	Controls	Controls	Controls
Province fixed effects	Controls	Controls	Controls	Controls	Controls
Observations	8,637	5,937	9,048	8,637	9,634
R2	0.764	0.555	0.571	0.554	0.638

Robust standard errors are in parentheses. *** *p* < 0.01, ** *p* < 0.05, * *p* < 0.1.

#### 4.2.3 Excluding the impact of financial markets on TFP

To avoid the interference of the financial crisis and government assistance policies for enterprises on the regression results, this study excludes samples from before 2011. The TFP calculated by the LP method is then re-regressed. As shown in the second column of Table 4, the regression coefficient of treatNCDP remains significantly negative, indicating that the research findings of this study are reliable.

#### 4.2.4 Extending the forecast window

To test the robustness of the core effects over a longer time horizon, we extended the original regression timeline and expanded the prediction window. In columns (3) and (4) of Table 4, the core dependent variables were lagged by one period and two periods, respectively. The results show that statistically significant outcomes persist even with an extended observational window, indicating that the negative impact of NCDP on enterprise TFP has long-term sustainability. This temporal misalignment approach helps mitigate causal identification issues to some extent, thereby reinforcing the robustness of the findings over extended timeframes.

#### 4.2.5 Substitution of dependent variables

To test robustness, we replaced the measurement methodology by calculating TFP using the Olley-Pakes (OP) and ordinary least squares (OLS) methods, as alternatives to the Levinsohn-Petrin (LP) approach. Columns (3) and (4) in Table 2, and column (5) in Table 4, present the regression results after substituting LP with OP and OLS methodologies. The outcomes are consistent with the baseline regression findings, confirming the robustness of the primary results across alternative TFP measurement frameworks.

#### 4.2.6 Placebo test

**Figure 4:**
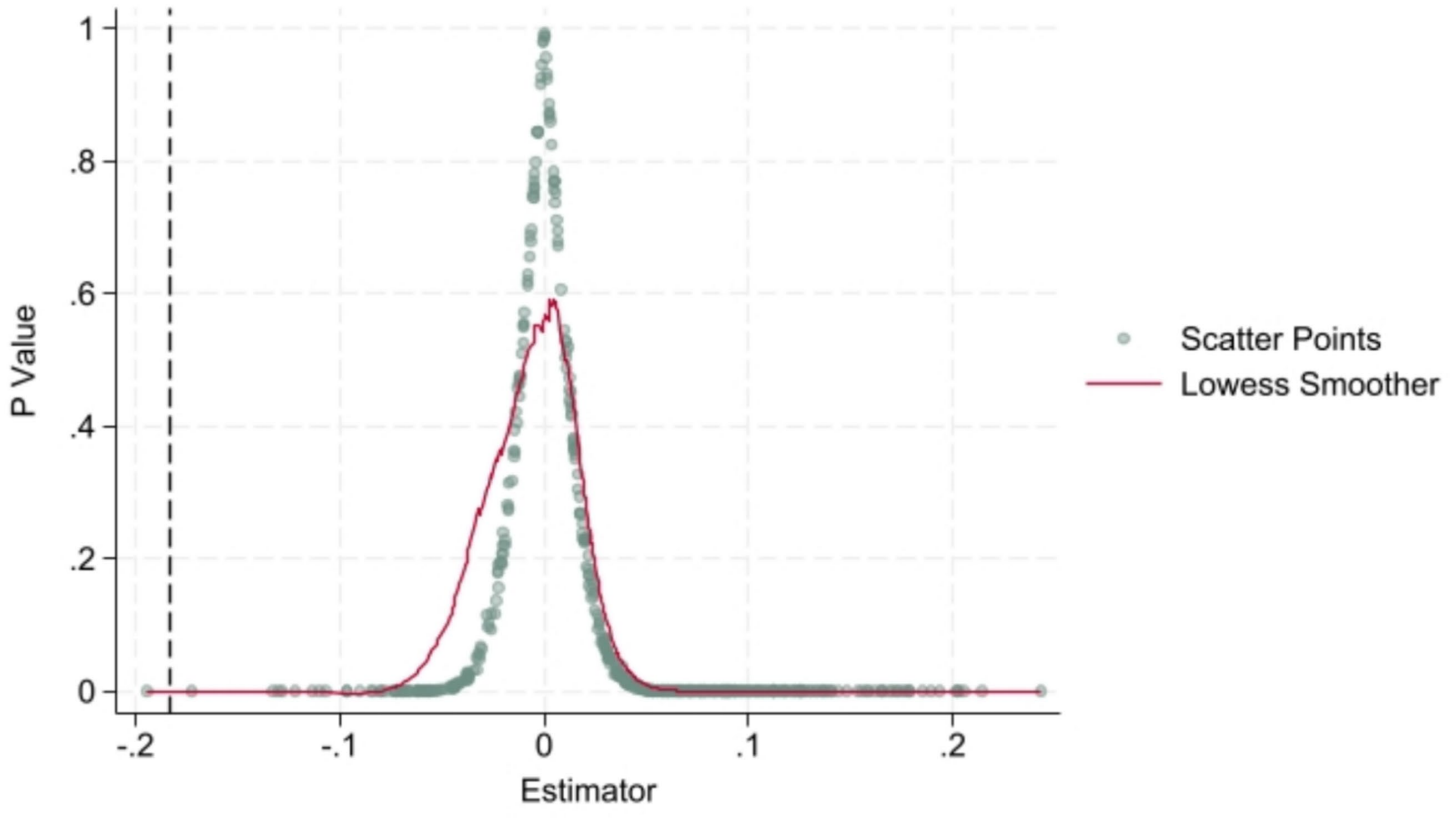
Placebo test results.

To verify that the observed changes in pharmaceutical manufacturers’ TFP are driven by the NCDP and not by unobserved confounding factors, we conducted a placebo test using randomized treatment group assignment. By performing 500 iterations of random sampling and regression, we generated kernel density estimates for the coefficient distribution of the key independent variable (Figure 4). The placebo test results show that the coefficients cluster around zero, with the majority being statistically insignificant. Only a small fraction of coefficients deviate below the true regression coefficient, providing strong evidence that the TFP changes in pharmaceutical enterprises are primarily attributable to the NCDP rather than random noise or external shocks.

### 4.3 Heterogeneity analysis

Previous empirical results show that the demand-side industrial policy, the NCDP, has reduced the TFP of pharmaceutical manufacturing enterprises. Demand-side industrial policies involve government interventions that replace market mechanisms to regulate corporate behavior. In this context, the impact of selective industrial policies may vary across firms with different characteristics. Therefore, if the decline in corporate TFP is attributable to NCDP implementation, this effect should vary across firms with different characteristics. Building on this, the study investigates the mechanisms through which NCDP affects TFP by examining cross-sectional differences in the impact of industrial policy shocks. It uses grouped regression methods, focusing on factors such as firm ownership, product characteristics, and analyst attention.

#### 4.3.1 Ownership-based examination

How does firm ownership influence TFP changes during NCDP implementation? To analyze the relationship between industrial policy and firm heterogeneity, Model (2) is specified. The grouping criterion differentiates state-owned enterprises (SOEs, coded SOE = 1) from non-state-owned enterprises (non-SOEs, coded SOE = 0).


(2) $$\begin{array}{l} \mathrm{TF}P_{\mathrm{it}}=\beta_{0}+\beta_{1}\text{treatNCDP}*+\beta_{2}\mathrm{SO}E_{\mathrm{it}}+\beta_{3}\text{contro}l_{\mathrm{it}}+\\ \sum \text{Time}+\sum \text{Prov}+\varepsilon_{\mathrm{it}} \end{array}$$

The grouping test results based on firm ownership are shown in columns (1) and (2) of Table 5. The regression results show a negative coefficient for non-state-owned enterprises (non-SOEs), indicating that the NCDP shock significantly reduced their TFP. The results further indicate that non-SOEs are more sensitive to industrial policy shocks than state-owned enterprises (SOEs). These findings suggest that the TFP-reducing effect of NCDP is more pronounced for non-SOEs than for SOEs.

**Table 5:** Heterogeneity analysis.

	Ownership heterogeneity		Product heterogeneity		Analyst coverage heterogeneity	
	(1) soe = 1	(2) soe = 0	(3) TCM = 1	(4) TCM = 0	(5) AnCov = 1	(6) AnCov = 0
	tfp_lp	tfp_lp	tfp_lp	tfp_lp	tfp_lp	tfp_lp
treatNCDP	−0.092 (0.071)	−0.268*** (0.045)	0.049 (0.064)	−0.246*** (0.043)	−0.348*** (0.060)	0.020 (0.044)
_cons	−3.729*** (0.523)	−4.612*** (0.318)	−8.979*** (0.446)	−2.879*** (0.309)	−3.775*** (0.550)	−3.216*** (0.274)
Controls	Yes	Yes	Yes	Yes	Yes	Yes
Time fixed effects	Controls	Controls	Controls	Controls	Controls	Controls
Province fixed effects	Controls	Controls	Controls	Controls	Controls	Controls
Observations	2,777	5,859	3,010	5,627	2,560	6,077
R-squared	0.649	0.516	0.654	0.521	0.504	0.585

Robust standard errors are in parentheses. *** *p* < 0.01, ** *p* < 0.05, * *p* < 0.1.

In practice, state-owned pharmaceutical manufacturers are primary suppliers of essential drugs to Chinese medical institutions, with government-appointed management and government-controlled entities at various levels. Their products inherently serve quasi-public welfare purposes rather than being purely profit-driven, with low profit margins and high production volumes. The NCDP’s primary aim—reducing drug prices through centralized procurement while ensuring supply volumes—aligns with the founding purpose of SOEs. As SOEs primarily produce high-volume, low-price drugs with stable demand, the policy has limited disruptive effects on their operations. Therefore, the policy’s impact on SOEs remains statistically insignificant.

From another perspective, governments, as bulk purchasers, have strong bargaining power to suppress prices and control accounts receivable, generally weakening supplier performance. However, this pressure diminishes when suppliers improve their own bargaining power. Compared to private firms, SOEs benefit from political affiliations, policy support, resource advantages, and strong financial capacities, which give them better negotiation power. This explains why the negative impact of customer concentration on performance is primarily observed in private enterprises. In the pharmaceutical industry, listed non-SOEs typically maintain higher profit margins. Participation in NCDP subjects their profitability to substantial shocks. As a result, non-SOEs experience greater declines in TFP than SOEs under this policy regime.

#### 4.3.2 Product heterogeneity-based examination

As a distinct category of pharmaceutical manufacturers in China, Traditional Chinese Medicine (TCM) producers hold a significant share in the national drug supply system. To empirically investigate whether differences in TFP exist between TCM and non-TCM manufacturers under the NCDP policy, this study uses the following Model (3). The grouping criterion distinguishes TCM manufacturers (coded as TCM = 1) from Western medicine manufacturers (coded as TCM = 0).


(3) $$\begin{array}{l} \mathrm{TF}P_{\mathrm{it}}=\beta_{0}+\beta_{1}\text{treatNCDP}*+\beta_{2}\mathrm{TC}M_{\mathrm{it}}+\beta_{3}\text{contro}l_{\mathrm{it}}+\\ \sum \text{Time}+\sum \text{Prov}+\varepsilon_{\mathrm{it}} \end{array}$$

The results are presented in columns (3) and (4) of Table 5. The regression results indicate that the coefficient for Western medicine manufacturers is negative and statistically significant, while the coefficient for TCM manufacturers is not significant. This confirms that the NCDP shock significantly reduces the TFP of Western medicine manufacturers but does not have a significant impact on TCM manufacturers.

Several factors contribute to this result. Firstly, TCM products lack a standardized national quality system, making it difficult to differentiate quality levels. The regulatory authorities have not yet established a comprehensive quality system for TCM formulations, limiting the basis for implementing a “quality-based pricing” management approach. Secondly, the production and pricing of TCM formulations are influenced by various factors. TCM formulations are derived from herbal medicines, which are agricultural products affected by factors such as region, climate, and environment. As a result, production supply is unstable and prices fluctuate frequently. Therefore, the prices of TCM products are traditionally regulated by market mechanisms and vary with market conditions. Lastly, the use of TCM formulations varies significantly among medical institutions. Different regions, hospitals, and medical practices use different TCM products, making it challenging to implement a nationwide NCDP model. Instead, smaller-scale procurement at the county level or within regional medical communities may be more appropriate. Given that TCM formulations were not included as primary targets in the NCDP, the policy does not significantly impact the TFP of TCM manufacturing enterprises.

#### 4.3.3 Analyst coverage-based examination

Securities analysts serve as important information intermediaries in capital markets. They can access information promptly, utilize their knowledge and analytical skills, generate and disseminate firm-specific information, reduce information asymmetry between internal (management) and external (investors) stakeholders, and improve capital allocation efficiency (46). When analysts anticipate a decline in firm performance, they may downgrade their ratings, prompting investors to react negatively, adversely affecting the firm’s stock price (47). Within the operational implementation of the NCDP policy, the inclusion of enterprises inevitably exerts profit-level impacts that disrupt business operations. To investigate whether differential market attention affects corporate TFP, this study employs Model (4), where pharmaceutical manufacturers under analyst coverage are coded as *AnCov* = 1 and those without analyst coverage as *AnCov* = 0:


(4) $$\begin{array}{l} \mathrm{TF}P_{\mathrm{it}}=\beta_{0}+\beta_{1}\text{treatNCDP}*+\beta_{2}\text{AnCo}v_{\mathrm{it}}+\beta_{3}\text{contro}l_{\mathrm{it}}+\\ \sum \text{Time}+\sum \text{Prov}+\varepsilon_{\mathrm{it}} \end{array}$$

Based on the heterogeneity of analyst attention, the results of the grouped test are presented in Columns 5 and 6 of Table 5. In the context of NCDP implementation, the policy inevitably affects the profitability of the included firms. The empirical results from the heterogeneity analysis indicate that TFP is indeed influenced by analyst coverage. Some literature suggests that when firms fail to meet analysts’ earnings forecasts, they may reduce future R&D investments, negatively impacting innovation (48). Therefore, under analyst coverage, NCDP negatively impacts enterprise TFP.

### 4.4 Mechanisms of influence test

#### 4.4.1 Financing constraints and TFP

The preceding analysis has established the relationship between the NCDP policy and the TFP of pharmaceutical manufacturers. The next step is to examine the mediating pathways through which NCDP influences TFP. Since NCDP impacts corporate operating performance, the resulting changes in financing constraints may disrupt investment activities such as in fixed assets and R&D, thus affecting productivity levels (49, 50). This study proposes the transmission pathway: NCDP → Financing Constraints → TFP, and empirically measures the role of financing constraints in this process.

To test the hypothesis regarding NCDP’s impact on financing constraints, this study employs the Kaplan-Zingales (KZ) index as a proxy. Following Wei et al. (18), we construct the KZ index using data from Chinese listed pharmaceutical companies to estimate the level of financing constraints (KZ) for each firm. A higher KZ value indicates greater financing constraints. Additionally, we investigate how financing constraints influence TFP through changes in specific financing channels. To this end, we establish a financing structure framework, substituting the mediating variable KZ with endogenous financing (endofund), exogenous financing (exdofund), and two exogenous financing channels: equity financing (sharefund) and debt financing (debtfund).

In defining variables, this study follows the approach of Lu et al. (51) to measure endogenous financing, using cash flow derived from adjusted net profit. This is deemed more appropriate as retained earnings, which represent internally generated funds, face fewer usage restrictions. Exogenous financing is measured by the net cash flow from financing activities as reported in the corporate cash flow statements. For current-period debt financing, we use the change in the sum of long-term loans and bonds payable under long-term liabilities in the balance sheet. Equity financing is measured as the change in the sum of common stock and capital surplus. To control for firm size effects, all four variables—endogenous financing, exogenous financing, debt financing, and equity financing—are scaled by the total assets of the listed companies for the corresponding period.

We construct the following mediating effect models: Mediating Effect Models (5, 6) for NCDP’s Impact on TFP via Financing Constraints:


(5) $$KZ_{\mathrm{it}}=\gamma_{0}+\gamma_{1}\text{treatNCDP}*+\gamma_{2}\text{contro}l_{\mathrm{it}}+\sum \text{Time}+\sum \text{Prov}+\varepsilon_{\mathrm{it}}$$

(6) $$\begin{array}{l} \mathrm{TF}P_{\mathrm{it}}=\beta_{0}+\beta_{1}\text{treatNCDP}*+\beta_{2}KZ_{\mathrm{it}}+\beta_{3}\text{contro}l_{\mathrm{it}}+\\ \sum \text{Time}+\sum \text{Prov}+\varepsilon_{\mathrm{it}} \end{array}$$

Mediating Effect Models (7, 8) for NCDP’s structural impact on TFP via financing constraints:


(7) $$\begin{array}{l} {\text{endofund}}_{\mathrm{it}}/{\text{exdofund}}_{\mathrm{it}}/\\ {\text{sharefund}}_{\mathrm{it}}/\;{\text{debtfund}}_{\mathrm{it}}=\gamma_{0}+\gamma_{1}\text{treatNCDP}*+\gamma_{2}\text{contro}l_{\mathrm{it}}+\\ \sum \text{Time}+\sum \text{Prov}+\varepsilon_{\mathrm{it}} \end{array}$$

(8) $$\begin{array}{l} \mathrm{TF}P_{\mathrm{it}}=\beta_{0}+\beta_{1}\text{treatNCDP}*+\beta_{2}(\begin{array}{l} {\text{endofund}}_{\mathrm{it}}/{\text{exdofund}}_{\mathrm{it}}/\\ {\text{sharefund}}_{\mathrm{it}}/\;{\text{debtfund}}_{\mathrm{it}} \end{array})+\\ \beta_{3}\text{contro}l_{\mathrm{it}}+\sum \text{Time}+\sum \text{Prov}+\varepsilon_{\mathrm{it}} \end{array}$$

Table 6 (columns 1–6) presents the empirical results of the mediating effect tests. The findings indicate that the NCDP policy reduces pharmaceutical manufacturers’ TFP by exacerbating their financing constraints. This result aligns with existing literature, which widely supports the idea that heightened financing constraints lead to declines in TFP (52–54), confirming conventional understanding.

**Table 6:** Impact mechanisms of financing constraints on TFP.

	(1)	(2)	(3)	(4)	(5)	(6)
	KZ	tfp_lp	endofund	exdofund	sharefund	debtfund
treatNCDP	0.736*** (0.094)	−0.050*** (0.013)	−0.008*** (0.003)	−0.013** (0.005)	−0.013*** (0.004)	−0.000 (0.003)
KZ		−0.141*** (0.008)				
_cons	−2.729*** (0.637)	−4.955*** (0.248)	−0.087*** (0.019)	0.033 (0.031)	0.064** (0.027)	−0.031 (0.021)
Controls	Yes	Yes	Yes	Yes	Yes	Yes
Time fixed effects	Controls	Controls	Controls	Controls	Controls	Controls
Province fixed effects	Controls	Controls	Controls	Controls	Controls	Controls
Observations	9,634	9,634	9,634	9,634	9,634	9,634
R-squared	0.197	0.610	0.257	0.077	0.097	0.064

Robust standard errors are in parentheses. *** *p* < 0.01, ** *p* < 0.05, * *p* < 0.1.

The coefficients for the impacts of endogenous financing (endofund), exogenous financing (exdofund), and equity financing (sharefund) in the financing structure are significantly negative: −0.008, −0.013, and −0.013, with significance levels of 1, 5, and 1%, respectively. These results suggest that the NCDP policy disrupts pharmaceutical manufacturers’ financing channels by reducing internal, external, and equity financing, thereby intensifying financing constraints. In contrast, the mediating effect of debt financing (debtfund) is statistically insignificant, implying that the NCDP does not reduce TFP through debt financing.

These findings can be interpreted as follows: When the NCDP exacerbates financing constraints, external funding becomes less accessible, and financing costs rise significantly. Firms are forced to rely on internal funds for fixed investments. However, due to reduced retained earnings and the large scale of required investments, internal funds alone are insufficient to meet investment needs. As a result, firms often forgo profitable investment opportunities, leading to resource misallocation and productivity losses (55). In conclusion, the NCDP policy ultimately reduces TFP by amplifying financing constraints for pharmaceutical manufacturers.

#### 4.4.2 R&D investment, inefficient investment, and TFP

Building on the theoretical analysis above, which suggests that industrial policies may influence corporate TFP through two primary channels—R&D investment and investment efficiency—this study constructs the following mediating effect framework to validate this transmission mechanism. First, we clarify the measurement methods for the core mediating variables. R&D investment (RD) is measured as the ratio of quarterly R&D expenditure to operating revenue for China’s A-share listed pharmaceutical firms. This metric, consistent with the definition of R&D expenditure in the Accounting Standards for Business Enterprises, effectively reflects firms’ sustained resource allocation to technological innovation activities (56). Inefficient investment (invest) is estimated using the residual method from Richardson’s (57) expected investment Model (9).


(9) $$\begin{array}{l} \text{inves}t_{\mathrm{it}}=\alpha_{0}+\alpha_{1}\mathrm{Siz}e_{\mathrm{it}-1}+\alpha_{2}\mathrm{Le}v_{\mathrm{it}-1}+\alpha_{3}\text{Growt}h_{\mathrm{it}-1}+\\ \alpha_{4}\mathrm{Ro}a_{\mathrm{it}-1}+\alpha_{5}\mathrm{Ag}e_{\mathrm{it}-1}+\alpha_{6}\mathrm{Cas}h_{\mathrm{it}-1}+\\ \alpha_{7}{\text{invest}}_{\mathrm{it}-1}+\sum \text{Time}+\sum \mathrm{Ind}+\varepsilon_{\mathrm{it}-1} \end{array}$$

Specifically, the dependent variable is defined as the ratio of net capital expenditures (net cash flow from the acquisition and disposal of long-term assets) to total assets at the beginning of the period. The independent variables include: $\mathrm{Siz}e_{\mathrm{it}-1}$, which represents the natural logarithm of total assets for firm i in period t − 1; $\mathrm{Le}v_{\mathrm{it}-1}$, denoting the leverage ratio, calculated as total liabilities divided by total assets; $\text{Growt}h_{\mathrm{it}-1}$, reflecting the total asset growth rate; $\mathrm{Ro}a_{\mathrm{it}-1}$, measuring return on assets, defined as net profit relative to total assets; $\mathrm{Ag}e_{\mathrm{it}-1}$, indicating firm age; and $\mathrm{Cas}h_{\mathrm{it}-1}$, capturing cash flow intensity, expressed as operating cash flow scaled by total assets. The residual term is denoted as $\varepsilon_{\mathrm{it}}$, with year and industry fixed effects controlled for. The absolute value of the regression residuals represents the degree of inefficient investment (INV), where positive residuals indicate over-investment and negative residuals reflect under-investment. This study takes the absolute value of the residuals for both over- and under-investment to construct INV, which captures the overall level of inefficient investment.

Building on this foundation, this study constructs a three-stage recursive model system to systematically examine the transmission mechanism. First, Model (10) investigates the impact of industrial policy (treatNCDP∗) on mediating variables (RD/INV). Model (11) tests the effect of mediating variables on TFP. Furthermore, to address potential chained mediating effects, Model (12) is introduced to analyze the transmission role of R&D investment on inefficient investment. The specific model specifications are as follows:


(10) $$\begin{array}{l} RD_{\mathrm{it}}/\;\text{IN}V_{\mathrm{it}}=\gamma_{0}+\gamma_{1}\text{treatNCDP}*+\gamma_{2}\text{contro}l_{\mathrm{it}}+\\ \sum \text{Time}+\sum \text{Prov}+\varepsilon_{\mathrm{it}} \end{array}$$

(11) $$\begin{array}{l} \mathrm{TF}P_{\mathrm{it}}=\beta_{0}+\beta_{1}\text{treatNCDP}*+\beta_{2}(RD_{\mathrm{it}}\;\text{IN}V_{\mathrm{it}})+\\ \beta_{3}\text{contro}l_{\mathrm{it}}+\sum \text{Time}+\sum \text{Prov}+\varepsilon_{\mathrm{it}} \end{array}$$

(12) $$\begin{array}{l} \text{IN}V_{\mathrm{it}}=\gamma_{0}+\gamma_{1}\text{treatNCDP}*+\gamma_{2}RD_{\mathrm{it}}+\gamma_{3}\text{contro}l_{\mathrm{it}}+\\ \sum \text{Time}+\sum \text{Prov}+\varepsilon_{\mathrm{it}} \end{array}$$

Through Models (10, 11), the independent mediating effects of R&D investment and investment efficiency can be identified. The inclusion of Model (12) further uncovers the “R&D investment → investment efficiency optimization → productivity enhancement” chain-mediated pathway, thereby systematically dissecting the multi-tiered mechanisms through which industrial policies influence corporate TFP.

According to the regression results in Table 7, the direct effect coefficient of NCDP on R&D investment is 0.009 and significantly positive, indicating that the policy effectively incentivizes corporate R&D activities. However, the results reveal that the direct effect coefficient of R&D investment on TFP is −0.133 and significantly negative, which contradicts the widely accepted view in the literature that “R&D investment enhances TFP growth.” To explain this paradox, a novel perspective is required: the productivity-enhancing effect of R&D investment exhibits a significant time lag, as technological transformation and market validation are needed to realize R&D outcomes (55, 58). In the short term, efficiency losses may arise due to resource crowding-out (32). This mechanism is further validated in our study—columns (3)–(5) of Table 6 demonstrate that the NCDP policy significantly increases corporate inefficient investment (INV), and this mediating effect through inefficient investment leads to a decline in TFP. Drawing on the theoretical framework of Czarnitzki and Hottenrott (59), the expansion of R&D investment under NCDP conditions may crowd out liquidity and productive capital for equipment renewal, resulting in a “R&D expansion-production contraction” resource allocation imbalance that exacerbates investment inefficiency. The empirical results also support this logic. Consequently, three mutually reinforcing mediating pathways are identified:

**Table 7:** Impact mechanisms of R&D investment and inefficient investment on TFP.

	(1)	(2)	(3)	(4)	(5)
	RD	tfp_lp	INV	tfp_lp	INV
treatNCDP	0.009*** (0.003)	−0.133*** (0.039)	0.006** (0.003)	−0.144*** (0.051)	0.009*** (0.003)
RD		−2.183*** (0.314)			0.123*** (0.032)
INV				−1.215*** (0.281)	
_cons	0.006 (0.009)	−4.638*** (0.245)	0.006 (0.020)	−3.899*** (0.287)	0.019 (0.018)
Observations	8,637	8,637	4,865	4,803	5,408
Controls	Yes	Yes	Yes	Yes	Yes
Time Fixed Effects	Controls	Controls	Controls	Controls	Controls
Province Fixed Effects	Controls	Controls	Controls	Controls	Controls
R-squared	0.528	0.557	0.179	0.583	0.183

Robust standard errors are in parentheses. *** *p* < 0.01, ** *p* < 0.05, * *p* < 0.1.

NCDP → RD↑ → TFP↓;

NCDP → INV↑ → TFP↓;

NCDP → RD↑ → INV↑ → TFP↓

#### 4.4.3 Financing constraints, R&D investment, and inefficient investment

Building on existing theoretical frameworks and supporting literature, this study posits that NCDP may influence TFP by exacerbating corporate financing constraints (KZ). These constraints, in turn, affect TFP through two distinct pathways: R&D investment (RD) and inefficient investment (INV). To validate this mechanism, the study constructs sequential regression models:

First, Model (13) is employed to examine the impact of NCDP on KZ:


(13) $$KZ_{\mathrm{it}}=\gamma_{0}+\gamma_{1}\text{treatNCDP}*+\gamma_{2}\text{contro}l_{\mathrm{it}}+\sum \text{Time}+\sum \text{Prov}+\varepsilon_{\mathrm{it}}$$

Subsequently, Model (14) is utilized to test the effects of financing constraints on the mediating variables RD and INV:


(14) $$\begin{array}{l} RD_{\mathrm{it}}/\;\text{IN}V_{\mathrm{it}}=\beta_{0}+\beta_{1}\text{treatNCDP}*+\beta_{2}KZ_{\mathrm{it}}+\beta_{3}\text{contro}l_{\mathrm{it}}+\\ \sum \text{Time}+\sum \text{Prov}+\varepsilon_{\mathrm{it}} \end{array}$$

The regression results in Table 8 reveal that, after controlling for time and province fixed effects, financing constraints (KZ) have a significantly positive direct effect on R&D investment (RD), with a coefficient of 0.011, and also exhibit a significantly positive coefficient of 0.007 on inefficient investment (INV). This seemingly paradoxical finding can be explained theoretically: on one hand, when firms face financing constraints, they may adopt a “flight-to-quality” strategy (60), increasing R&D investments to build technological barriers in exchange for capital market recognition or policy preferences. On the other hand, credit resource crowding-out forces firms to reduce strategic investments and pursue short-term high-risk projects, leading to deteriorating investment efficiency (61). The coexistence of these two pathways suggests that financing constraints exert a dual effect on corporate behavior, driving both “innovation incentives” and “investment distortions.” The underlying drivers of these effects can be traced to policy-driven expectations of R&D subsidies (59) and resource substitution behaviors induced by credit rationing (62). Consequently, this study identifies two competing transmission channels, with the net effect of industrial policies on productivity determined by the interplay of these opposing mechanisms.

**Table 8:** Impact mechanisms of financing constraints on R&D investment and investment inefficiency.

	(1)	(2)	(3)
	KZ	RD	INV
treatNCDP	0.736*** (0.094)	0.011*** (0.003)	0.007*** (0.003)
KZ		−0.003*** (0.000)	0.002*** (0.000)
_cons	−2.729*** (0.637)	0.007 (0.009)	0.027 (0.019)
Controls	Yes	Yes	Yes
Time fixed effects	Controls	Controls	Controls
Province fixed effects	Controls	Controls	Controls
Observations	9,634	8,446	4,744
R-squared	0.197	0.541	0.186

Robust standard errors are in parentheses. *** *p* < 0.01, ** *p* < 0.05, * *p* < 0.1.

NCDP → KZ↑ → RD↑ → TFP↑,

NCDP → KZ↑ → INV↑ → TFP↓

## 5 Conclusion

### 5.1 Research findings

This study empirically examines the impact of the National Drug Price Control Policy (NCDP) on TFP of pharmaceutical firms using quarterly data from A-share listed pharmaceutical manufacturing companies in China from 2003 to 2021. The results show that NCDP significantly hinders the improvement of corporate TFP, with the negative impact being most pronounced in non-state-owned enterprises, non-traditional Chinese medicine producers, and firms with high analyst coverage. Mechanistically, NCDP directly suppresses TFP by exacerbating financing constraints (KZ↑), which manifest as reductions in internal, external, and equity financing capabilities, thus diminishing capital allocation efficiency. In addition, financing constraints indirectly affect TFP through two channels: on one hand, they push firms to increase R&D investments (RD↑), but the time lag in innovation conversion prevents these efforts from translating into immediate productivity gains; on the other hand, they lead to inefficient investments (INV↑), causing capital misallocation and efficiency losses. While R&D expenditures rise in the short term, they result in a “resource crowding-out and substitutive squeezing effect,” which, in turn, worsens investment efficiency and amplifies the negative impact on TFP through the “NCDP→RD↑ → INV↑ → TFP↓” transmission chain. These findings suggest that, while NCDP offers public welfare benefits by reducing drug costs, its negative effects on corporate financing capacity, investment efficiency, and forced R&D escalation may undermine the long-term development of firms, creating a trade-off between “policy dividends and enterprise efficiency.”

### 5.2 Policy implications

To balance the public welfare objectives of NCDP with the long-term sustainability of pharmaceutical companies, the following policy recommendations are made based on the empirical findings:

First, establish a categorized support system and multi-tiered financing safeguards. Policy design should be tailored to address the diverse impacts of NCDP across firms. For non-state-owned enterprises, non-traditional Chinese medicine producers, and firms with high analyst visibility that are disproportionately affected by the policy, differentiated compensation mechanisms should be incorporated into price control policies. These could include relaxing loan access restrictions, streamlining equity issuance approvals to reduce external financing constraints, and enhancing internal financing capacity through tax rebates and retained earnings subsidies. Additionally, strengthen policy-driven financial tools by introducing industry-specific credit quotas for pharmaceuticals, exploring supply chain finance and intellectual property pledge financing models, and guiding private capital into innovative drug R&D to address the “financing difficulty → low investment efficiency → R&D resource crowding-out” dilemma.

Second, align innovation incentives with endogenous corporate reforms. In terms of R&D support, policy should include proportional VAT and income tax deductions for corporate R&D expenditures, phased subsidies for clinical trials of innovative drugs, and the establishment of patent commercialization reward funds to mitigate resource displacement risks. Furthermore, equity incentive schemes for core R&D personnel should link compensation to innovation outcomes, boosting human capital returns. In corporate governance, firms should optimize capacity structures by divesting low-efficiency assets and focusing on high-margin product lines to enhance capital allocation efficiency. Investment decision-making processes should be refined through dynamic project evaluation and risk-warning systems to mitigate TFP erosion caused by inefficient investments. Finally, the creation of a dynamic NCDP policy monitoring platform should be considered to periodically assess the relationship between price controls and productivity fluctuations. This platform would help achieve long-term equilibrium between “price containment and efficiency enhancement” through flexible pricing mechanisms and phased compensation policies.

## Data Availability

Publicly available datasets were analyzed in this study. This data can be found at: https://data.csmar.com/.
